# Supplementary material for: Content-rich biological network constructed by mining PubMed abstracts
Source: BMC Bioinformatics. 2004 Oct 8;5:147. doi: 10.1186/1471-2105-5-147 (PMC528731; doi:10.1186/1471-2105-5-147)
Supplement: Additional File 2 — The original results of the above study (non-essential files are deleted to keep the file size under the limit set by BMC bioinformatics). [file 1471-2105-5-147-S2.bz2 › chilibotAdditionalFile2/dip20/753ID9852089E18138/html/DNAA_DNAB.html]

 


 **DNAA** and **DNAB** 
  
Found 99 abstracts in PubMed, retrieved 20.  
 

 What does Google say? 
 PDF only 
| .edu only 

---

**Interactive relationship** (e.g. stimulation, inhibition, etc)

**Neutral relationship**- The interaction domains of the  **DnaA**  and  **DnaB**  replication proteins of Escherichia coli.  Ref: 10972842 Mol Microbiol, 2000
- Escherichia coli  **DnaA**  protein loads a single  **DnaB**  helicase at a  **DnaA**  box hairpin.  Ref: 12161435 J Biol Chem, 2002
- We propose that contacts between  **DnaA**  and  **DnaB**  occur via two interaction sites on each of the proteins.  Ref: 10972842 Mol Microbiol, 2000
- We show that  **DnaA**  protein actively mediates the entry of  **DnaB**  at oriC.  Ref: 9852089 J Biol Chem, 1998
- Other evidence suggests that proline 28 of  **DnaA**  is involved in the recruitment of  **DnaB**  to oriC.  Ref: 12864864 Mol Microbiol, 2003
- We defined physical contacts between  **DnaA**  and  **DnaB**  that involve residues 24 86 and 130 148 of  **DnaA**  and residues 154 210 and 1 156 of  **DnaB**  respectively.  Ref: 10972842 Mol Microbiol, 2000
- Size exclusion chromatography, surface plasmon resonance, and electron microscopy experiments demonstrated that the  **DnaB**  helicase is delivered to the  **DnaA**  box region, which is localized approximately 200 base pairs upstream from the region of origin opening and a potential site for helicase entry.  Ref: 11316803 J Biol Chem, 2001
- **DnaA**  was distributed throughout the cytoplasm, but both  **DnaB**  and DnaI were always detected as foci during the cell division cycle.  Ref: 10844689 Mol Microbiol, 2000
- That only two  **DnaB**  hexamers are bound, one for each replication fork, suggests that the binding of additional molecules of  **DnaA**  in forming the prepriming complex restricts the loading of additional  **DnaB**  hexamers that can bind at oriC.  Ref: 11551962 J Biol Chem, 2001
- The initiation of chromosome replication in Escherichia coli requires the recruitment of the replicative helicase  **DnaB**  from the DnaBC complex to the unwound region within the replication origin oriC, supported by the oriC bound initiator protein  **DnaA** .  Ref: 10972842 Mol Microbiol, 2000
- The  **DnaA**  replication initiation protein has been shown to be essential for DNA strand opening at the AT rich region of the replication origin of the Escherichia coli chromosome as well as serving to recruit and position the  **DnaB**  replicative helicase at this open region.  Ref: 12835421 Proc Natl Acad Sci U S A< MedlineTA>Proc Natl Acad Sci U S A, 2000

**Non-interactive relationship** (e.g. studied together, co-existance, homology, etc.)

- Thus, the TrfA 44 initiation protein has the multifunctional properties of recruiting and positioning an active form of the  **DnaB**  helicase at the RK2 replication origin by a  **DnaA**  independent process.  Ref: 12835421 Proc Natl Acad Sci U S A< MedlineTA>Proc Natl Acad Sci U S A, 2000
- Stoichiometry of  **DnaA**  and  **DnaB**  protein in initiation at the Escherichia coli chromosomal origin.  Ref: 11551962 J Biol Chem, 2001
- We report here that the Escherichia coli replication proteins  **DnaA** , which is required to initiate replication of both the chromosome and plasmid pSC101, and  **DnaB** , the helicase that unwinds strands during DNA replication, have effects on plasmid partitioning that are distinct from their functions in promoting plasmid DNA replication.  Ref: 10601213 J Bacteriol, 1999
- This is based on the competition between the lambda P protein and the host  **DnaA**  and DnaC proteins for  **DnaB**  helicase.  Ref: 11429468 Microbiology, 2001
- The AT rich region could be unwound by E. coli  **DnaA**  protein, and the  **DnaB**  helicase was loaded into the single stranded bubble.  Ref: 11606418 EMBO Rep, 2001
- In the prepriming complex, the stoichiometries are 10  **DnaA**  monomers and 2  **DnaB**  hexamers per oriC plasmid.  Ref: 11551962 J Biol Chem, 2001
- Although the origin opening caused by binding of the host  **DnaA**  protein has been shown to be critical for the loading of the  **DnaB**  helicase, to date there has been no direct evidence presented for the formation of the  **DnaB**  complex at the  **DnaA**  box site.  Ref: 11316803 J Biol Chem, 2001
- Since  **DnaA**  induces a local unwinding of oriC, one model is that the limited availability of single stranded DNA at oriC restricts the number of  **DnaB**  molecules that can bind.  Ref: 12161435 J Biol Chem, 2002
- High levels of  **DnaA**  protein in the presence of the TrfA protein also resulted in the stimulation of open complex formation and  **DnaB**  helicase loading on oriV, even in the absence of the four  **DnaA**  boxes.  Ref: 10417640 Mol Microbiol, 1999
- Strand specific loading of  **DnaB**  helicase by  **DnaA**  to a substrate mimicking unwound oriC.  Ref: 12421318 Mol Microbiol, 2002
- As was observed previously for  **DnaA** , overexpression of the wild type  **DnaB**  protein conversely stabilized the inheritance of partition defective plasmids while not increasing plasmid copy number.  Ref: 10601213 J Bacteriol, 1999
- The Escherichia coli PriA protein loads the  **DnaB**  replicative helicase at branched DNA structures independently of the replication initiator protein,  **DnaA** , and thereby facilitates assembly of functional replisomes at sites removed from oriC.  Ref: 12581361 Mol Microbiol, 2003
- Although, interaction of  **DnaA**  with  **DnaB**  could not substitute for RepA  **DnaB**  interaction for helicase loading,  **DnaA**  along with integration host factor, DnaC, and RepA was essential for helicase loading.  Ref: 9874774 Proc Natl Acad Sci U S A, 1999
- However, as shown in this study, TrfA 44 specifically interacts with the  **DnaB**  replicative helicase of Pseudomonas Pseudomonas putida and Pseudomonas aeruginosa and initiates the formation of a prepriming open complex in the absence of  **DnaA**  protein.  Ref: 12835421 Proc Natl Acad Sci U S A< MedlineTA>Proc Natl Acad Sci U S A, 1999
- In E.coli DnaT and DnaC are responsible for loading  **DnaB**  onto the DNA during primosome assembly, in the PriA and  **DnaA**  mediated cascades, respectively.  Ref: 11842108 Nucleic Acids Res, 2002
